# Pulmonary Squamous Cell Carcinoma With Hemopneumothorax Related to Pleuroparenchymal Fibroelastosis Post-Liver Transplantation

**DOI:** 10.1016/j.atssr.2025.07.017

**Published:** 2025-08-20

**Authors:** Shinya Otsuka, Haruhiko Shiiya, Hideki Ujiie, Nanase Okazaki, Yoshihiro Matsuno, Tsuyoshi Shimamura, Masaaki Watanabe, Junichi Nakamura, Satoshi Tanaka, Tatsuya Kato

**Affiliations:** 1 Department of Thoracic Surgery, Hokkaido University Hospital, Sapporo, Japan; 2 Department of Surgical Pathology, Hokkaido University Hospital, Sapporo, Japan; 3 Division of Organ Transplantation, Hokkaido University Hospital, Sapporo, Japan; 4 Department of Transplant Surgery, Faculty of Medicine and Graduate School of Medicine, Hokkaido University, Sapporo, Japan; 5 Department of Respiratory Medicine, Faculty of Medicine, Hokkaido University, Sapporo, Japan; 6 Center for Cause of Death Investigation, Faculty of Medicine, Hokkaido University, Sapporo, Japan

## Abstract

Pleuroparenchymal fibroelastosis (PPFE) is a rare interstitial pneumonia subtype. Its association with lung cancer remains unclear, with limited reported cases. We present a case of non-small cell lung cancer (NSCLC) with PPFE after liver transplantation. Eleven years post-transplant, the patient experienced hemopneumothorax, requiring emergency surgery, and received a diagnosis of NSCLC. Although cancer recurrence was not shown, the patient died of an acute exacerbation of interstitial pneumonia that had spread from both lower lobes 12 months later. In lung cancer with PPFE, considering treatment strategies with careful monitoring of life-threatening complications such as hemopneumothorax and interstitial pneumonia exacerbation is crucial for long-term survival.

Pleuroparenchymal fibroelastosis (PPFE) is a rare subtype of interstitial pneumonia (IP) that manifests as an upper lung field lesion.^1^ Reports of PPFE complicated by lung cancer, particularly squamous cell carcinoma (SqCC), are limited, and its pathologic and clinical features remain unclear. Here we describe a case of lung SqCC accompanied by lung PPFE in a patient after liver transplantation.

A 56-year-old man underwent deceased donor liver transplantation for liver failure resulting from alcoholic liver disease. The patient had a 600 pack-year smoking history, but he quit smoking after liver transplantation. Immunosuppressant agents and corticosteroids were administered after transplantation, but only tacrolimus was continued, because the side effects of the other drugs. Four years after liver transplantation, reticular shadows were observed in the left and right upper lobes, suggesting PPFE. The interstitial lung abnormalities showed slow progression. However, at that time, no treatment modality with solid evidence had been established for PPFE; therefore, in consideration of the potential risk of pneumothorax associated with steroid therapy, we opted for a strategy of clinical observation. Eight years after liver transplantation, a pulmonary cyst was found inside the reticular shadow. Moreover, a nodule was observed on the cystic wall. The nodule gradually progressed, and lung cancer was suspected (Figure 1). Although surgical treatment was planned 11 years after liver transplantation, the patient experienced hemopneumothorax and underwent emergency lobectomy. Gross examination of the resected left upper lobe of the lung revealed a subpleural pulmonary cyst approximately measuring 43 mm, with a 4-mm pleural perforation. In addition, a 17-mm solid mural nodule, histologically nonkeratinizing SqCC (pT2a N0 M0 stage IB) accompanied by PPFE, was found not only invading the cystic wall and surrounding lung tissue, but also showing extensive in situ spread along the inner surface of the cyst. Squamous metaplasia was not evident in epithelium lining the cystic wall or surrounding the lesion. Immunohistochemical examination showed no programmed death-ligand 1 expression, and loss of nuclear p53 protein expression, a so-called null pattern suggesting nonsense, frameshift, or splice-site, mutations of *TP53* gene (Figure 2). Hemosiderin deposition was limited, which is inconsistent with the progression of chronic bleeding, and acute intracystic hemorrhage was suggested.

**Figure 1:**
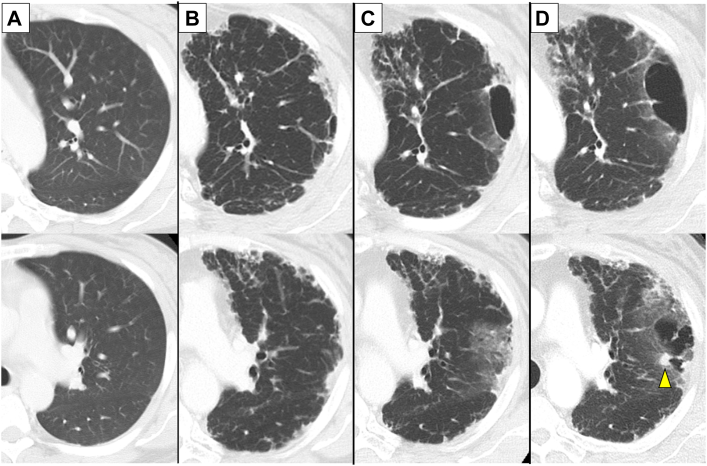
Time-course changes on computed tomographic images of the left upper lobe. (A) No significant findings were shown in the recipient’s lung at the time of liver transplantation. (B) A subpleural reticular shadow and consolidation were indicated 9 years after liver transplantation. (C) A pulmonary cyst appeared in the reticular shadow 12 years after transplantation. (D) The cyst had enlarged, and a solid nodule (arrowhead) was observed adjacent to the cyst 12 years and 8 months after transplantation.

**Figure 2:**
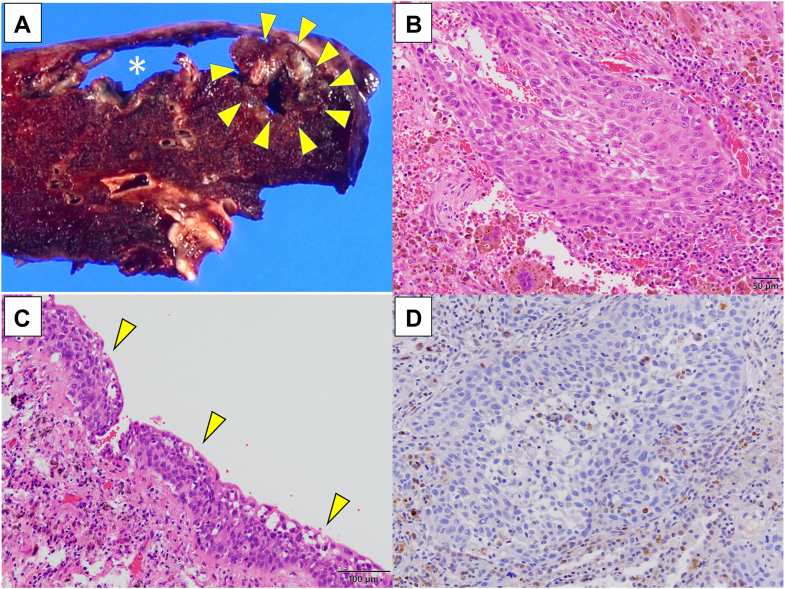
Macroscopic and microscopic images of the resected left upper lobe of the lung. (A) On the cut surface, a subpleural large pulmonary cyst (asterisk) and a solid tumor invading the cyst wall (arrowheads) were shown. (B) Histologic examination of the solid mural nodule revealed nonkeratinizing squamous cell carcinoma. (Hematoxylin and eosin; original magnification ×200.) (C) Wide extension of carcinoma in situ (arrowheads) was found along the inner surface of the cyst wall. (Hematoxylin and eosin; original magnification×100.) (D) Immunohistochemistry showed loss of nuclear p53 protein expression (null pattern) in tumor cells. (Anti-p53 antibody [DO7]; original magnification ×100.)

Postoperatively, the tacrolimus dose was gradually reduced, and the patient received everolimus for its antioncogenic effect. He did not receive adjuvant chemotherapy because of the risk of infection. Ten months after lobectomy, ground-glass shadows suggestive of nonspecific IP were detected in the left and right lower lobes. Given the rapid enlargement of ground-glass opacities, steroid pulse therapy and intravenous cyclophosphamide were administered (Supplemental Figure). Nevertheless, his respiratory status deteriorated, and he died of acute exacerbation of IP 12 months after lung surgery. The autopsy revealed PPFE in the apex of the right lung (Figure 3) and a non–usual IP (UIP) pattern and diffuse alveolar damage in the lower part.

**Figure 3:**
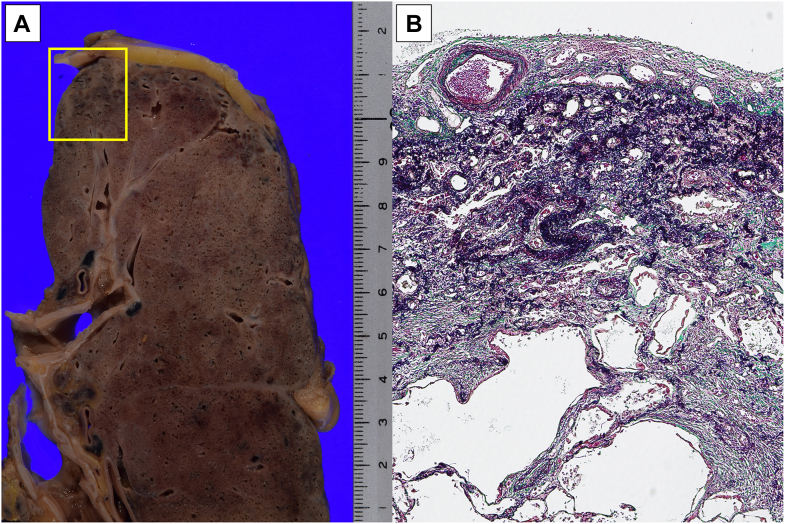
Images of the right lung at autopsy. (A) Macroscopic findings of the right lung. The yellow square indicates the area shown in Figure 3B. (B) Subpleural fibrosis with thickening of the alveolar septum and proliferation of elastic fibers were observed in the apex of the lung. These findings were consistent with pleuroparenchymal fibroelastosis. (Elastica Van Gieson; magnification ×200.)

## Comment

In this case, computed tomography revealed subpleural reticular shadowing and consolidation in the upper lobes of both lungs. Histopathologic examination of the same region at biopsy showed subpleural fibrosis with thickening of the alveolar septum and proliferation of elastic fibers, confirming the diagnosis of PPFE.

Reports on the coexistence of PPFE and lung cancer, particularly SqCC, are limited, and the pathologic link remains unclear.^2^ The patient’s lung cancer development was likely influenced by his smoking history and posttransplant immunosuppression. Chronic inflammation and fibrosis from IP may activate p53, leading to cellular senescence and transforming growth factor-β pathway activation, thereby contributing to lung cancer progression.^3^ However, immunohistochemical analysis in this case suggested p53 function loss, leaving its role in IP progression uncertain.

Although only 1 report exists of PPFE in a recipient’s lungs after liver transplantation,^4^ the mechanism of PPFE after liver transplantation has not been clarified because of the small number of cases and the lack of detailed genetic investigation.

In this case, hemopneumothorax developed, requiring emergency surgery. Pneumothorax associated with PPFE may result from strong inspiratory effort causing high transpulmonary and transluminal pressures and is reported in 25% to 89% of PPFE cases.^5^ Although pathologic findings did not suggest tumor perforation of the pleura in this patient, enlargement and fragility of the cyst may have been influenced by tumor progression. High transpulmonary pressure and acute intracystic hemorrhage could contribute to the development of hemopneumothorax. In lung cancer with PPFE, careful monitoring for pneumothorax and hemopneumothorax risk is essential.

Although postoperative recurrence of lung cancer was not observed, the patient died of the progression of IP with a non-UIP pattern, which had spread from both lower lobes. The prognosis of patients with PPFE could be worse in those with lower lobe UIP,^6^ but acute exacerbation carries a potentially fatal risk even in patients without non-UIP. Therefore, timely therapeutic intervention is crucial to achieving long-term survival.

In lung cancer with PPFE, considering treatment strategies with careful monitoring of life-threatening complications such as hemopneumothorax and IP exacerbation is key for long-term survival.

## Figures and Tables

**Supplementary Figure:**
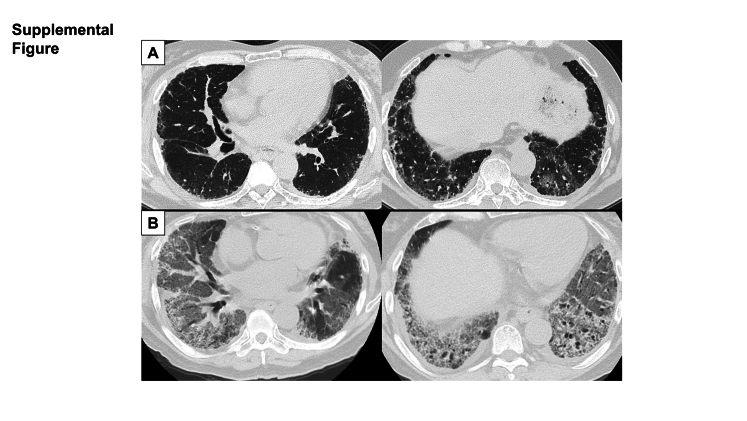
Computed tomography findings of lower-lobe interstitial pneumonia. (A) Ground-glass opacities were observed in both lower lobes 10 months after lobectomy. (B) At 12 months post-lobectomy, ground-glass opacities had further increased, accompanied by progression of traction bronchiectasis.
