# Supplementary material for: Pulmonary Squamous Cell Carcinoma With Hemopneumothorax Related to Pleuroparenchymal Fibroelastosis Post-Liver Transplantation
Source: Ann Thorac Surg Short Rep. 2025 Aug 20;4(1):248–51. doi: 10.1016/j.atssr.2025.07.017 (PMC13100806; doi:10.1016/j.atssr.2025.07.017)
Supplement: Supplementary Figure Legend [file mmc1.docx]

**Supplemental Figure Legends**

Supplemental Figure. Computed tomography findings of lower-lobe interstitial pneumonia.

A) Ground-glass opacities were observed in both lower lobes 10 months after lobectomy.

B) At 12 months post-lobectomy, ground-glass opacities had further increased, accompanied by progression of traction bronchiectasis.
